# Parents’ pandemic NICU experience in the United States: a qualitative study

**DOI:** 10.1186/s12887-021-03028-w

**Published:** 2021-12-09

**Authors:** Ashlee J. Vance, Kathryn J. Malin, Jacquelyn Miller, Clayton J. Shuman, Tiffany A. Moore, Annella Benjamin

**Affiliations:** 1 National Clinician Scholars Program, University of Michigan, School of Nursing, NCRC Building 14, Suite G-100, 2800 Plymouth Road, Ann Arbor, MI 48109 USA; 2 Marquette University, College of Nursing, Milwaukee, WI USA; 3 Institute for Healthcare Policy and Innovation, University of Michigan, Ann Arbor, USA; 4 University of Michigan, School of Nursing, Ann Arbor, USA; 5 University of Nebraska Medical Center, College of Nursing, Omaha, NE USA; 6 University of Michigan, Ann Arbor, USA

**Keywords:** Parents, COVID-19, Neonatal intensive care unit, NICU, Qualitative

## Abstract

**Background:**

Prior to the COVID-19 pandemic, parents of infants in the Neonatal Intensive Care Unit (NICU) frequently reported high levels of stress, uncertainty, and decreased parenting confidence. Early research has demonstrated that parents have had less access to their infants in the hospital due to restrictions on parental presence secondary to the pandemic. It is unknown how parents have perceived their experiences in the NICU since the beginning of the COVID-19 pandemic. The purpose of this study was to describe the lived experience of parents who had an infant in the NICU in the context of the COVID-19 pandemic to inform healthcare providers and policy makers for future development of policies and care planning.

**Methods:**

The study design was a qualitative description of the impact of the COVID-19 pandemic on parents’ experiences of having an infant in the NICU. Free-text responses to open-ended questions were collected as part of a multi-method study of parents’ experiences of the NICU during the first six months of the pandemic. Participants from the United States were recruited using social media platforms between the months of May and July of 2020. Data were analyzed using a reflexive thematic approach.

**Findings:**

Free-text responses came from 169 parents from 38 different states in the United States. Three broad themes emerged from the analysis: (1) parents’ NICU experiences during the COVID-19 pandemic were emotionally isolating and overwhelming, (2) policy changes restricting parental presence created disruptions to the family unit and limited family-centered care, and (3) interactions with NICU providers intensified or alleviated emotional distress felt by parents. A unifying theme of experiences of emotional distress attributed to COVID-19 circumstances ran through all three themes.

**Conclusions:**

Parents of infants in the NICU during the first six months of the COVID-19 pandemic experienced emotional struggles, feelings of isolation, lack of family-centered care, and deep disappointment with system-level decisions. Moving forward, parents need to be considered essential partners in the development of policies concerning care of and access to their infants.

**Supplementary Information:**

The online version contains supplementary material available at 10.1186/s12887-021-03028-w.

## Background

The COVID-19 pandemic created unprecedented conditions for administrators and clinicians working in Neonatal Intensive Care Units (NICU) and greatly affected parents of infants requiring hospitalization. Prior to the COVID-19 pandemic, parents of infants admitted to a NICU reported high levels of stress, anxiety, uncertainty, and decreased parenting confidence when compared to parents of healthy full-term infants [1–6]. Approximately 28–40% of mothers of infants admitted to a NICU were diagnosed with a new mental illness, such as depression or perinatal post-traumatic stress disorder [7]. Fathers of infants requiring NICU hospitalization also reported significant stress and need for reassurance and support [8, 9].

Adverse parental mental health associated with NICU admissions affects parent-infant bonding, parental physical health, and infant cognitive development outcomes [10–14]. Studies have shown that when an infant requires NICU hospitalization, the normative transition to parenthood can be altered, resulting in worsened parental mental health and confidence [15, 16]. For this reason, many hospitals have implemented family-centered care practices to help mitigate the disruption of the transition to parenthood [17–19] and provide unrestricted access to their hospitalized infant to optimize neurodevelopmental outcomes and parent mental health [20–22].

Like many aspects of life prior to the pandemic, parenting and family life were exceptionally susceptible to unanticipated changes during the COVID-19 pandemic, potentially resulting in elevated levels of stress and uncertainty [23, 24]. Subsequently, when families experienced an infant’s admission to the NICU, this likely resulted in further exacerbation of stress. While there is sufficient evidence demonstrating the negative parental outcomes secondary to having an infant hospitalized in the NICU prior to the COVID-19 pandemic, there is little qualitative data regarding how parents have experienced infant hospitalization during the COVID-19 pandemic. Recent reports document a decrease in parental presence by 32% and participation in rounds by 30% in the United States [25]. Globally, 52% of parents from 56 different countries reported restricted access to their infants while hospitalized in the NICU, with more restrictive policies being associated with reported worry by parents [26]. Moreover, these restrictions to parental presence are associated with decreased bonding and negatively impact breastfeeding [27]. Several commentaries have called attention to visitor practices and downstream effects of new COVID-19 policies, such as risk for moral distress, fear for safety and injury to providers [28, 29] and increased risk to infant and family well-being [30]. Yet, to date there are few studies describing the parent experience of COVID-19-related policies and the parent experience navigating infant hospitalization during the COVID-19 pandemic.

Accordingly, we sought to understand the NICU experience from parents’ perspectives in order to provide a more comprehensive and detailed understanding of the experiences and needs of families. Our aim was to describe the lived experience of parents, who had an infant hospitalized in a Neonatal Intensive Care Unit in the context of the COVID-19 pandemic during the first wave of infection in the United States. This research was conducted to serve all NICU healthcare providers and policy makers in order to inform future decisions regarding supporting families in the NICU.

## Methods

We used a qualitative descriptive design to analyze open-ended, free-text data collected as part of a larger multi-method study to describe parents’ experiences of NICU hospitalization during the COVID-19 pandemic [31]. Subjects were recruited between the months of May – July 2020 using social media platforms such as NICU parent support groups, Facebook, Twitter, and Instagram. Parents were eligible for participation if they had an infant requiring NICU hospitalization between February 1, 2020 – July 31, 2020. These dates were chosen as eligibility criteria to ensure the data captured would reflect the parenting experience during the first six months of the COVID-19 pandemic [32]. An anonymous online survey was developed using the Research Electronic Data Capture (REDCap) research database [33]. The survey included questions about parent demographics, infant health, and hospital-related characteristics; family, social, and NICU environments; several validated measures related to parent experience; and five open-ended questions. The study was deemed exempt by the Institutional Review Board at the University of Michigan and is reported in accordance with the Journal Article Reporting Standards for Qualitative Research [34]. Participants did not receive compensation for participation.

Participants were asked to respond to five open-ended questions concerning the impact of the COVID-19 pandemic on the experience of having a baby in the NICU, the birth experience, the transition home, interactions with healthcare providers, and parental presence experience (see Supplementary Materials). The free text responses were exported from REDCap to a document that was imported to NVivo 11 [35]. An organic, descriptive, thematic analysis was employed to identify shared patterns of meaning-making in parents’ experiences in the NICU during COVID-19 [36]. Our approach was constructivist in that we centered parents’ experiences while acknowledging the investigators’ role in interpreting and synthesizing those experiences in the present thematic description [37]. A sociologist with qualitative expertise (JM) led the coding and analysis with regular consultation and discussion with the study team, which was composed of nurse scientists with expertise in parenting, stress, and the NICU.

Analysis followed the steps of reflexive thematic analysis [36], beginning with immersion in the data through rereading. During a first round of open coding, topic and category codes were developed inductively, though informed by the investigators’ prior NICU research (for example, we anticipated the important topics would likely include emotion experience, staff interactions, and uncertainty). The team then generated tentative themes through reorganizing, consolidating, prioritizing, and mapping codes and categories, followed by a second round of coding focused on these thematic areas (for codebook, see Supplementary Material). Next, we checked initial themes against the overall dataset, considering alternative explanations and outliers. Finally, we named, defined, and described the themes through discussion and analytic memo writing [38].

## Results

Of a total of 178 online survey respondents, 169 answered at least one of the five open-ended questions (94.9%). Respondents lived in 38 states in the United States and 97% identified as the mother (*n* = 164). Parental, infant, and hospital characteristics are provided in Table 1. Answers to questions ranged in length from a phrase or sentence to a full page. Some parents reported facts impassively while others elaborated on how they felt about them; some volunteered information on topics not specifically solicited, such as work and finances.

**Table 1:** Parent, Infant, and Hospital Characteristics

*Parent Characteristics*	
	Mean (SD)
Age (years)	31 (5.4)
Number of individuals in home	3.8 (1.3)
	**n (%)**
Total respondents	169 (100)
Race/ethnicity	
Asian, American Indian, Hawaiian	3 (2)
non-Hispanic Black	4 (2)
non-Hispanic White	105 (62)
non-Hispanic More than one race	4 (2)
Hispanic/Latinx	13 (8)
Not answered	40 (24)
Number of children	
1	79 (47)
2–3	68 (40)
4 or more	22 (13)
Marital status	
Not partnered	9 (5)
Married/Living with partner	119 (70)
Not answered	41 (24)
Education	
Some high school	4 (2)
High school graduate/GED	16 (9)
Some college or 2-year degree	37 (22)
College graduate	40 (24)
Graduate/Professional degree	32 (19)
Not answered	40 (24)
Income	
< 35,000	27 (16)
35,000 – 75,000	41 (24)
> 75,000	58 (34)
Not answered	44 (26)
Insurance	
Private insurance	89 (53)
Government health plan	38 (22)
No coverage	2 (1)
Not answered	40 (24)
*Infant Characteristics*	
	**Mean (SD)**
Age at time of survey (weeks)	9.7 (6.9)
Length of NICU stay (days)	41 (31)
	**n (%)**
Total respondents	169 (100)
Gestational category	
Extremely preterm (23–27)	37 (22)
Preterm (28–33)	74 (44)
Late preterm (34–36)	25 15
Term (> 37)	35 (21)
Not answered	23 (14)
Reason for admission	
Prematurity	119 (70)
Congenital birth defects	6 (4)
Neurological	12 (7)
Other	31 (18)
Not answered	1 (1)
*Hospital-Related Characteristics*	
	**Mean (SD)**
Hours with infant in NICU per day	7.7 (6.1)
	**n (%)**
Total respondents	169 (100)
Distance to hospital	
Less than 30 min	64 (38)
30–60 min	41 (24)
1–2 h	14 (8)
More than 2 h	9 (5)
Not answered	41 (24)
Frequency of visitation	
Daily	115 (68)
Every couple days	8 (5)
Weekly	4 (2)
As schedule allowed	2 (1)
Not answered	40 (24)
Video visitation with infant	
No	89 (53)
Yes	40 (24)
Not answered	40 (24)
Hospital type	
NICU in a children’s hospital	47 (28)
Community hospital with a NICU	49 (29)
NICU at an academic medical center/regional care center	27 (16)
Observational/small special care nursery	1 (1)
Unsure/Other	5 (3)
Not answered	40 (24)

**Fig. 1:**
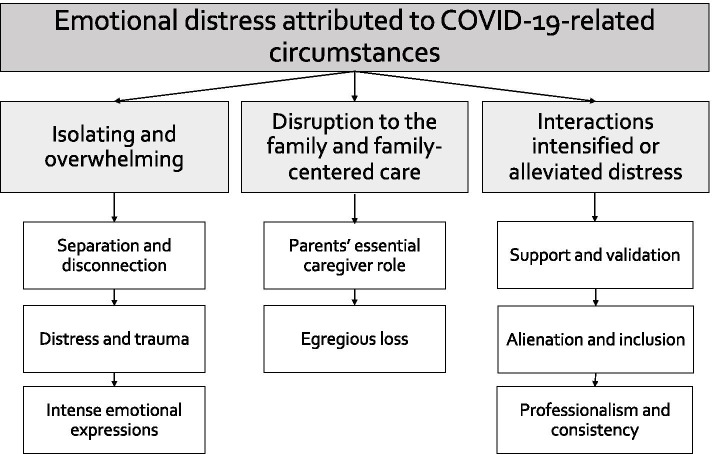
Themes and Subthemes

We began examining the dataset for continuities and discontinuities between parents’ experiences in the NICU during the COVID-19 pandemic above and beyond non-pandemic times. Through this lens, we developed three broad themes: (1) parents’ NICU experiences during the COVID-19 pandemic were emotionally isolating and overwhelming, (2) policy changes restricting parental presence created disruptions to the family unit and limited family-centered care, and (3) interactions with NICU providers intensified or alleviated emotional distress felt by parents. Exemplar quotes representing these three themes are displayed in Table 2. A unifying theme running through all three themes was experiences of emotional distress attributed to COVID-19- related circumstances (see Fig. 1).

**Table 2:** Themes, Subthemes, and Exemplar Quotes

Theme 1: Parents’ NICU experiences during the COVID-19 pandemic were isolating and overwhelming	
*Subtheme: Isolation and disconnection*	
*“My child almost did not make it and It was hard because I was the only one aloud in the NICU and I was alone to cry and didn’t have the emotional support.”* (White mother of twins from Maine) *“My wife and I had to lean on each other instead of leaning on family and friends. We not only had a son, but we also lost one, so it was an emotional roller coaster.”* (White mother of twins from California) *“We never imagined not having our families and closest friends be with us during this amazing time in our lives*.” (White mother of 1 from New Jersey) *“Both my husband and I were unable to visit our baby at the same time, and no other visitors were allowed. Doing this alone was tough and took a huge toll on my mental state.”* (Black mother of 4 from Pennsylvania) *“Being the only one who could visit my daughter was incredibly difficult and taxing. My husband and I very much intend to share parenting roles, but it was not possible for him to participate in person at all while she was in the NICU. We definitely understand the precaution, but it was so taxing on both me and him.”* (White Mother of 1 From North Carolina)	
*Subtheme: Distress and trauma*	
*“This was my 2nd child to be in the NICU. My first child was there in 2017. While that was stressful, the added impact of COVID made this time much lonelier.”* (White mother of 2 from Texas) *“[Our son] died unexpectedly with no real warning before his crisis. Due to covid we have very few pictures of him without a mask over our faces and none with his older sister. The NICU isn’t easy in the best of times but when a child dies in the middle of the pandemic you can’t have the normal support of friends and family due to isolating to stay safe for your remaining child.”* (White mother of 3 from Colorado) *“It was extremely challenging. Our son being born prematurely was already a traumatic experience and now we were not allowed to be together to visit him in the NICU.”* (Father of 1 from California, unknown race/ethnicity) *“Hospital policies not in touch with lives reality of families making the impossible pain of baby in NICU even more impossible.”* (White mother of 1 from Washington) *“It is hard to discern which emotions are related to losing our other son* vs. *the NICU stay* vs. *COVID -- so, I will just comment that it could be a combo of the above impacting my responses. It has been hard to mourn our son [twin A] we lost in the midst of a pandemic and taking care of a preemie [twin B*].” (White mother of preterm twins from California) *“It has been extra stressful because of COVID 19. The isolation is difficult especially [with] post-partum depression and OCD.”* (White mother of 2 from Kansas)	
*Subtheme: Intense emotional expressions*	
*“It’s scary coming and going home.”* (White mother of 5 From Texas) *“It was harder to feel like I was safe to love on my baby.”* (White mother of 3 from Wisconsin) *“We were terrified they were going to say we couldn’t visit at some point.”* (Mother of 3 from Iowa, unknown race/ethnicity) *“I had to deal with my anxiety, panic attacks, and talking with all the doctors and nurses completely alone and my husband couldn’t see his son for 5 weeks.”* (Mother of 2 from Tennessee, unknown race/ethnicity) *“We were sent home quickly after I [delivered], our baby stayed, and it broke my heart daily to leave him*.” (White mother of 3 from Texas) *“Everything is much scarier. I needed more family support, and I didn’t get that [because] me and dad were not allowed in the NICU together and that made me feel so alone. I want to share experiences with him and I couldn’t.* (White mother of 2 from Kansas) *“Only one parent was [able to] speak in person with all doctors, nurses, support teams, which then put so much on my shoulders. I was sad and trying to keep myself together. There were times I would forget what I wanted to ask or the information became so overwhelming, then having to come home and repeat all of this to dad was heartbreaking all over again.”* (Hispanic mother of 3 from New York)	
Theme 2: Disruption to the family and family-centered care	
*Subtheme: Parents’ essential caregiver role*	
*“The hospital as an institution put in place policies meant for the greater good, and yet seemingly not taking into account something that nurses and doctors have long practiced and preached: that family support of a baby in the neonatal intensive care unit is, in fact, essential.”* (White mother of 3 from Texas) *“He should he considered an essential caregiver and not a visitor. It’s been incredibly hard to handle everything on my own and cruel.”* (Mother of 2 from Tennessee, unknown race/ethnicity) *“Parents are not and should NOT be considered visitors. We are essential for the baby’s health and all of us need to be together as a family. Separating us in our already fragile state was incredibly stressful. We also need to be present to advocate for our baby. Moms and babies should be considered one unit, and Moms do need their partners for support.”* (Asian mother of 1 from Michigan) *“When only one parent was allowed, my son was very unstable, and we had to go through hearing some hard news without the support of our partner.”* (Hispanic motherof 1 from New Mexico) *“Since only one parent was allowed to visit at a time and we lived an hour and half away, my husband only got to visit our baby in the NICU 3 times in the 4 weeks he was there. He also didn’t get to meet or visit with any of the doctors, specialist or surgeons that our son was in the care of or hear anything firsthand about his diagnosis or treatment.”* (Mother of 5 from Texas, unknown race/ethnicity) *“Parents are being forced to be apart, spouses are missing weeks and weeks of their newborns lives, all while the child is exposed to numerous doctors, nurses, specialists, therapists,* etc. *This nonsensical policy [one parent at a time] has resulted in so much trauma and heartache.”* (Mother of 1 from Michigan, unknown race/ethnicity)	
*Subtheme: Egregious loss*	
Loss of bonding *“Bonding with our newborn as parents was a joke. My husband and I couldn’t be with our daughter at the same time. Many firsts were missed by one or the other*.” (Mixed-race mother of 1 from Utah) *“My husband and I would switch out every 12 h. This meant we didn’t spend any time with each other at home because we wanted someone to be with our daughter at all times…. It ruined our relationship because we could not be together seeing our baby.”* (Asian mother of 1 from Texas) *“Other parent had to wait until baby was discharged to meet the baby. Siblings were not able to meet the baby and bond. It became harder for siblings to understand what was happening.”* (Hispanic mother of 3 from New York) Loss of experiences *“Unfortunately, with no warning signs, my son died. Due to covid no one other than his father and I got to meet him for his 30 days of life. His older sister and grandparents only got to see pictures or videos. It was hard to leave the NICU since those were the only people who knew him … We have very few pictures of him without a mask over our faces and none with his older sister.”* (White mother of 3 from Colorado) *“I want to exclusively breastfeed and once my baby starts oral feeds only, he will have to take a bottle for most of his feeds because I cannot be there due to limited visiting hours. I am actually pretty upset about this, as I am worried, he will prefer the bottle over my breast.”* (White mother of 1 from Michigan) *“Only my husband and I are allowed and never at the same time, so no one else has visited our baby. We must always wear masks, so my baby can’t see our faces or hear our voices well. I can’t be in the same room as my husband and child at the same time. I can’t kiss my baby.”* (Mixed-race mother of 2 from Georgia) *“With face masks required by all we are worried our daughter will not know facial expressions and emotion.”* (White mother of 1 from Florida) *“We’re required to wear masks the entire time so I dislike how my child cannot see me smile or receive a kiss.”* (White mother of 1 from Texas) *“I get a disconnect with my daughter having to wear a mask every time I was able to visit with her.”* (White father of 2 from North Carolina) Loss of time *“Only I was allowed to see my baby for the first month. No family and not even baby’s dad was allowed in unless I was going to stay away for 24 h.”* (White mother of 3 from Wisconsin) *“We had to visit separate from each other, meaning our first time completely together as a family was 18 days later when our son came home.”* (White mother of 1 from Georgia) *“If my husband lives with me and I’m allowed in the NICU then he should be too … I can’t drive myself, so my husband drives me every day and he sits in the parking lot for the hours that I’m there.”* (Mother of 2 from Tennessee, unknown race/ethnicity) *“My husband and I could not visit our son in NICU at the same time. Having just given birth, I was not able to drive so my husband would drive me to the hospital while I visited, nursed, and pumped, while he had to wait in the car and then I would wait in the car while he came in.”* (White mother of 1 from North Carolina) *“We live together, eat together – in fact my husband was dropping me and picking me up from the hospital, so we are already exposed to each other, how does this reduce the risk?”* (Asian mother of 1 from Michigan)	
Theme 3: Interactions with NICU providers intensified or alleviated emotional distress	
*Subtheme: Support and validation*	
*“We understood the precautionary steps the NICU was taking, but maybe being a little more sensitive to what the parents are going through during those times may have helped.”* (Father of 1 from California, unknown race/ethnicity) *“The doctors could have been more sympathetic. The nurses could have been more supportive of our time with our child. They often do care time before or after his time, despite us coming specifically to help. They also sometimes discourage us holding him to let him rest. Because we already spend so little time with him, I feel sacrificing a bit of sleep would be worth it for the benefits he would experience being close to us.”* (Mixed-race Mother of 2 from Georgia) *“I felt as though no one understood the real trauma and pain I was dealing with having 2 infants in the NICU during uncertain times. I really struggled with leaving my babies and have never had NICU babies before my twins. I was struggling emotionally and mentally and didn’t feel like the doctors cared. I also felt like hospital policies were so strict they were causing unnecessary mental health issues on new parents with NICU babies. I am now in EMDR therapy to try to help process the loss and grief of our NICU stay & birth experience/trauma.”* (White mother of 4 from Washington)	
*Subtheme: Alienation and inclusion*	
*“They started doing their rounds* via *FaceTime. We mostly saw the nurses and nurse practitioners. We continuously told them we wanted a primary care team for our daughter, but they did not do that, mostly because they would continue switching out staff, so one person could not stay with her for too many days due to COVID.”* (Asian mother of 1 from Texas) *“Changing protocols 2–3 times a day. Staff was advised to have only minimum contact with all patients (*i.e.*, no lactation support, undiagnosed and untreated postpartum depression* etc.*).”* (Hispanic mother of 1 from Ohio) *“The biggest thing that bothers me is that the rotation of staff who work with our daughter is very random. She’s been here 27 days and has had more than 27 different nurses working with her. I hate this for many reasons, the first is that the more people she comes into contact with the higher her exposure risk. The other biggest reason is that I want someone invested in her care, someone who has gotten to know her like I have.”* (White mother of 2 from California) *We learned a lot about caring for our child and have used the skills at home with him. The doctors always took time to visit us daily, but sometimes it is hard to know what you do not know -- or what to ask?* (White mother of twins from California) *I’m not sure [what staff could do differently], unless the doctors could have more strongly advocated with the hospital for parental presence. They would say things like ‘do skin to skin is very helpful x number of times a day’ or ‘breastfeed x number of times a day’ seemingly without taking into account there were actually hospital policies making that very difficult or without following through in any helpful way to facilitate that*.” (White mother of 3 from Texas)	
*Subtheme: Professionalism and consistency*	
*“It was interesting to see the changing protocols, almost daily, as the hospital navigated the safest screening parameters for the both the maternity levels of the hospital and the NICU. I feel like the care we received was not impacted, everyone was incredibly professional.”* (White mother of twins from California) *“Some nurses wouldn’t wear a mask and face shield together (which was required) every time they came in the room which bothered me. Many touched their masks and touched my infant. Many pulled down their masks when they needed to catch their breath instead of walking out of the room, but I didn’t feel like I could speak up about it.”* (White mother of 2 from Colorado) *“Lack of information, changing protocols, general paranoia amongst nurses did not support confidence in the team to the point we did not dare to leave NICU fearing not being allowed to enter again.”* (Hispanic mother of 1 from Ohio) *“It was unnerving to be in the same hallway as covid babies. I did not like when I would see my nurse have to gown for one patient and then come into our room. It was also difficult to hear staff talk about going out on weekends while we were quarantined as much as possible to keep our baby safe.”* (White mother of 1 from Florida)

## Theme 1: parents’ NICU experiences during the COVID-19 pandemic were emotionally isolating and overwhelming

Over half of the parents wrote about the emotional and mental impacts of having an infant in the NICU during the COVID-19 pandemic. The isolating and overwhelming theme of parent experiences was exemplified by three subthemes: (1) isolation and disconnection, (2) distress and trauma, and (3) intense emotional expressions.

*Isolation and disconnection.* One of the most prevalent emotional and mental experiences described was that of isolation and disconnection:

*“I was alone. I had absolutely no family beside my new baby. It was one of the worst experiences I’ve ever had*.” (Mother of 2 from Michigan, race/ethnicity unknown).

Isolation and disconnection were attributed to various impacts of COVID-19 such as visitor restrictions; requirements to wear masks and gloves; the inability to kiss, hug, and touch infant; frequent staff turnover; and reduced or remote-only interactions with staff. Although related in cause and circumstance, isolation, loneliness, and separation were described in emotionally painful terms whereas feelings of disconnection were more often described as alienating, strange, and cold (see Table 2).

*Distress and trauma.* Another primary emotional experience was the sheer stress, difficulty, and overwhelming nature of the situation. Parents used expressions such as, “extremely difficult,” “awful,” “impossible,” “traumatizing,” and “brutal.” One parent wrote, “this experience has been the worst of my life” (White mother of 2 from Tennessee), another said, “it was a horrible experience, and I would never wish it on anyone” (Asian mother of 1 from Texas), and a third wrote, “this is another level” (Mixed-race mother of 2 from Georgia). Some parents reflected consciously on how much of their experience was due to the added stressors of the pandemic. While having an infant in the NICU is already stressful, many parents felt that the COVID-19 experience compounded the NICU stress to a high degree:

*“COVID made difficult situations even more difficult as we had restrictions accessing NICU. These restrictions made my relationship with my wife (baby mother) also difficult.”* (White father of 3 from Maryland).

*Intense emotional expressions.* There were many emotions reported by parents that ranged from grief, sadness, and anxiety to uncertainty, heartbreak, and fear*.* Parents’ descriptions of fear ranged from worry and anxiety to panic. Almost half the mentions of fear were related to COVID-19 in the NICU. Other fears were of losing visitor access, the infants’ well-being, worries about breastfeeding, and general anxiety from the stressors described above. Uncertainty due to changing pandemic-related policies also contributed to fear:

*“We felt very much out of control and in constant fear of not knowing.” (Black mother of 1 from Louisiana).*

Grief over losses such as infant death and debility are not unusual in the NICU, but more unique to the pandemic were expressions of sadness and heartbreak due to family separations, limited visits, or lost experiences with the infant resulting from COVID-19 policies.

*“Visitations were always bittersweet because one parent or the other wasn’t able to be there.”* (Mixed-race mother of 1 from Utah).

Although the present analysis focuses on parents’ experiences while their infant was in the NICU, it is important to consider the holistic experience of isolation parents described during and after their time in the NICU. COVID-19 restrictions often required mothers to be in the hospital with few or no visitors before, during, and after the birth, sometimes for weeks at a time. Numerous mothers emphasized that their birth experiences during COVID-19 were “scary” and “lonely.” Several mothers discussed their physical limitations of not being able to drive or move easily, which compounded their struggles with pumping, breastfeeding, or getting to the NICU. After the infant’s discharge from the NICU, parents described having limited in-person contact with family and friends due to concerns about spreading COVID-19 infection. This broader context of isolation likely colored parents’ experiences and memories of time in the NICU. This quote poignantly expresses the strange alienation of one mother’s experience of giving birth without the anticipated connection and community.

*“I feel like I missed out on a lot of the joys of pregnancy and birth because of the pandemic. Somehow don’t have any pictures of me pregnant, had no baby shower, no friends come meet the baby, no hospital visitors. Didn’t get to share the joys of it with family and friends. It’s almost like it all didn’t happen except for the fact there is now a baby hanging around.”* (White mother of 1 from New Jersey).

## Theme 2: disruption of the family and family-centered care

The second major theme was that NICU policies regarding parental presence disrupted the family unit instead of prioritizing infant and family-centered developmental care. Overall, parents expressed the strongest emotions about the direct and indirect impacts of policies restricting parental presence during the COVID-19 pandemic. Some parents reported confusion because of frequently changing policies. Among the more restrictive policies were those that restricted all parental presence in the NICU, only one designated parent/caregiver for the NICU stay, or switch-offs between parents/caregivers weekly. The most common visitation policy, reported by 77 parents (46%), was parental presence was limited to one parent/caregiver at a time.

Within this family theme, there are two subthemes. First, restrictive policies undermined parents’ role as essential to the infant’s caregiving team. Second, parents felt the resultant conflicts and losses as egregious, and much of their grief, isolation, overwhelm, confusion, and anger centered around these policies which they perceived as limiting family-centered care.

*Parents’ essential caregiver role.* Many parents implied, and seven explicitly stated, that visitation restrictions denied their ability to serve as essential caregivers for their infant.

*“I believe we should be seen as members of the care team and not visitors.”* (Mixed-race mother of 2 from Georgia).

While some parents accepted the policies as a regrettable necessity, others believed they were contradictory and even “nonsensical” (Mother of 1 from Michigan, unknown race/ethnicity). A few expressed their objections in the strongest terms as a violation of their parental rights.

*“My husband didn’t see his child for over a month. Which I feel is incredibly wrong. How can someone deny a parent access to their own child?”* (White mother of 1 from North Carolina).

*“My husband and I felt that our rights as parents were violated. My husband and I should have both been able to see our daughter. Instead, I was alone in the NICU. It caused us a great deal of trauma and pain. Not to mention that our daughter lost out on essential bonding time with her father.”* (Mixed-race mother of 3 from Arizona).

The new policies regarding parental presence impacted the direct care parents were able to provide. Parents reported constraints on breastfeeding and skin-to-skin care, which, they noted, healthcare providers cannot provide.

*“As the mother I had to continuously pump around the clock as well as do her cares (diapering, feeding) and then trying to find time to hold her to do skin-to-skin but that wasn’t possible because I didn’t have an extra set of hands that could help while I pumped. The nurses would take care of 3 babies at a time so they weren’t able to help out as much either, or they would prioritize other babies.”* (Asian mother of 1 from Texas).

Restricted access also interfered with their ability to advocate for their infant and for both parents to communicate with specialists. When policies restricted parental presence to one designated caregiver only, parents prioritized birth mothers remaining in the NICU, especially if they were breastfeeding or if the other parent needed to work, given concerns for employment security during the pandemic. This situation not only excluded partners from caregiving, advocacy, and learning opportunities, it also placed extra burdens on mothers.

*“The hospital policy changed so only one parent was allowed in the NICU at a time. This resulted in me receiving difficult news with no spouse or support person; making decisions about baby’s care without baby’s father present; myself physically navigating the NICU and interacting with baby without physical support even while I was recovering from delivery and had a broken rib.”* (White mother of 3 from Texas).

Sometimes physically and emotionally fragile after birth, mothers provided care and advocacy alone without in-person practical or emotional support from family. The mother would sometimes not receive any wellness breaks and were burden with the added strain of needing to relay critical information to the absent partner.

*Egregious loss.* In addition to interfering with parents’ functional role as essential caregivers, parents described restrictive parental presence policies that resulted in experiences of egregious loss. Descriptions of loss are summarized in three main ways: (1) loss of bonding, (2) loss of experiences, and (3) loss of time (see Table 2). Due to masking requirements, opportunities to hold, touch, or kiss infants were reduced. Parents worried that the lack of visible smiles and other facial expressions would impact infant development. Masks and visitation restrictions also interfered with photos for celebrating the family unit and introducing infants to family members.

Parents also felt the loss of opportunities for bonding between the infant and the second parent or sibling. This loss was attributed not only to the reduced time each family member could spend with the infant, but the loss of experiences that parents and siblings could share together as a family unit. For example, parents grieved not being able to share infants’ first holding or bathing. Three parents reported that the separations, differential burdens, and loss of shared experiences strained their marital relationship. The most restrictive policies regarding parental presence forced parents to make painful and difficult choices about care and being with their infant. In describing lengths of time separated from infants, parents often used modifiers like *only, just, still, over, never until,* and *whole* to express egregiousness, or by using extremely precise counts of days or hours. Parents used such expressions for both long and relatively brief time periods, suggesting that any length of separation could be experienced painfully.

## Theme 3: interactions with NICU providers intensified or alleviated emotional distress

The last theme identified was that NICU staff could either exacerbate or mitigate parents’ emotional strain. The significant role of healthcare providers in this theme is exemplified in three main subthemes: (1) support and validation, (2) alienation and inclusion, and (3) professionalism and consistency.

*Support and validation.* Parents voiced a deep need and desire for sympathy, acknowledgement, and support from the NICU providers to validate the reality of their difficult experience. There was a striking contrast between reports of parents who felt staff acknowledged the extreme difficulty of the NICU during the COVID-19 context and those who did not perceive that acknowledgement. Parents who received sympathetic recognition found it validating and supportive, while those who did not found this discordance with staff added to their burden.

*“The nurses were AMAZING. I felt empowered, comforted, respected, and cared for. There was a general understanding that NICU is hard enough but then adding COVID made it even worse. That felt validating.”* (White mother of 1 from North Carolina).

*“Really great team and very caring. They all empathized with us knowing this is unprecedented times.”* (White mother of 1 from Texas).

*“They didn’t seem to acknowledge that it’s a very difficult time to have a baby in NICU never mind during the pandemic. Some nurses were mean and nowhere near as supportive as they should have been. A couple of nurses were AMAZING. Some doctors were also harsh and only seemed to see us as another number and not humans needing individual care.”* (Mixed-race mother of 3 from California).

*Alienation and inclusion.* Parents described feeling alienated or isolated from the caregivers responsible for the care of their infants. This was attributed to COVID-19 changes including exclusion of parents from rounds, remote consults, perceptions of staff shortages, high staff turnover, reduced parent services (e.g., lactation support, mental health support), social distancing, and masking.

*“It was hard to recognize faces of hospital staff (doctors, nurses, techs, ect) with masks which made things feel more distant.”* (White mother of 1 from North Carolina).

Some parents were concerned that turnover compromised infant care through reducing staff familiarity with the infant as well as increasing the number of contacts and potential COVID-19 exposures. Others reported that due to restricted access to staff, their ability to advocate for their infant was significantly hindered. Several parents wanted to feel more included either in care or in giving feedback to the hospital system. Parents wanted an opportunity to express feelings concerning policies restricting parental presence; inconsistencies between recommendations, policies, and practices; and their desire to participate more in their infants’ care.

*“We really wish the hospital involved parents in such decision making, it would have avoided a lot of unnecessary trauma.”* (Asian mother of 1 from Michigan).

*Professionalism and consistency.* Parents reported intense discomfort with inconsistent adherence to COVID-19 precautions on the part of some NICU staff, as well as with a lack of professionalism in staff conversations about infant care or changing policies.

*“[Nurses were] talking loudly about each baby’s care and their parents, discussing what they did and didn’t agree with about Covid changes. Sometimes touching hair, face, ect with gloves on and not changing them.”* (Black mother of 1 from New Mexico).

Some parents were concerned that turnover compromised infant care through reducing staff familiarity with the infant as well as increasing the number of contacts and potential COVID-19 exposures.

## Discussion

The purpose of this study was to critically examine the experience of parents of infants admitted to a NICUs during the first six months of the COVID-19 pandemic. We acknowledge the unprecedented circumstance of the COVID-19 pandemic and the rapid responses required to maintain safety for the most vulnerable patients in times of extreme uncertainty. We also acknowledge the lack of evidence-based solutions for policies regarding parental presence in the NICU during the early months of the pandemic. Even so, the descriptions provided by parents during their infant’s hospitalization during this time provides valuable insight into this challenging situation and offers strategies to improve care. Our results add to the growing body of knowledge surrounding the detrimental impact of restrictions on parental presence in the NICU [39, 40].

Overwhelmingly, parents described their experience of neonatal hospitalization through painful expressions of separation, disconnection, and isolation. These descriptions are similar to other accounts of experiences under pandemic restrictions in other populations [41, 42]. Importantly, the qualitative descriptions provided by parents in our study demonstrate that vulnerable populations, such as parents of infants requiring hospitalization, call for unique consideration when providing healthcare in the face of uncertainty and rapid change. Previous research has established that parents who experience feelings of isolation, stress, and uncertainty are more likely to suffer from poor mental health after their time in the hospital [43, 44]. These outcomes have long-term consequences for the health of parents as well as that of infants [10].

These findings are striking given that providing infant and family-centered developmental care and supporting healthy transitions into parenthood are fundamental to the care provided in the NICU [45]. Consistency in care is especially important during periods of transition. Maternal-infant healthcare providers are ideally positioned to recognize the unique and emerging needs of the families they serve. When families experience frequent turnover from care providers while in the NICU, there is a loss of trust and increased feelings of abandonment [18]. Parent health must be prioritized when allocating clinical resources in the NICU during times of rapid changes. During times of great uncertainty, purposeful assessment of parents’ needs is required so that resources, such as psychology and nursing support, can be tailored accordingly. Shared decision making should be emphasized, and parents represented in development of policies and procedures when possible. Additionally, parents themselves offer a strategy to mitigate feelings of loss of control and uncertainty, by valuing them as “essential care” [46]. If parental presence in the NICU must be limited secondary to legitimate concerns for safety, more resources like psychology services, strong family advisory boards, and personalized care planning must also be prioritized.

**Fig. 2:**
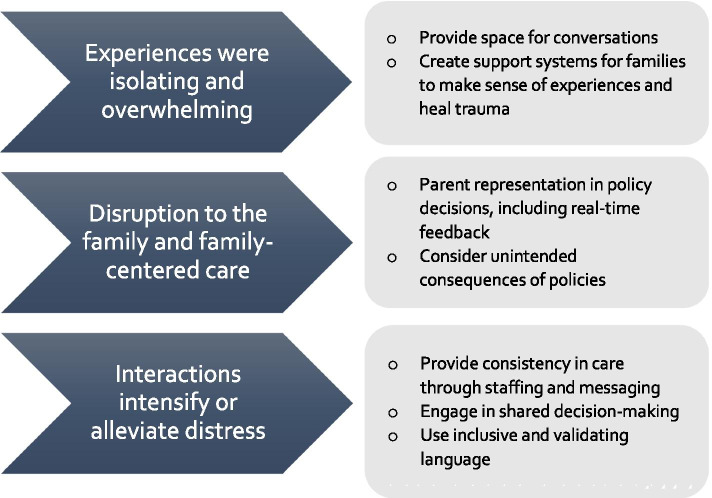
Themes and related potential interventions

Our findings indicate parents of hospitalized infants during the COVID-19 pandemic report overwhelming emotional strain, splitting the family unit, and a deep need for more NICU staff support. As the effects of the COVID-19 pandemic continue to reverberate throughout our society, it is important to consider the unintended injury that families of hospitalized infants experienced during this time. Healthcare providers must recognize the unique needs of these families during exceptional circumstances and adjust in the support that is provided. Furthermore, there are lessons to be learned from these parents’ reports. First, providing space and creating systems that allow families to tell their stories can help the healing of the trauma experienced from having a hospitalized neonate. Researchers and healthcare providers are charged with supporting parents through their processing as well as facilitating parenting skills and adaptation [47]. Second, providing consistency in care, through both staffing and messaging, is a low-effort and high-impact method to support families. The remarkable fact, found in both in our study and previous research, is that parents of hospitalized infants do not need healthcare providers to have all the answers, rather they need to be heard and supported [48, 49]. Parental perceptions of illness are crucial to the health of the family [5]. Having a consistent model of care to support families as they make sense of their situation and plan for the future is imperative. This is even more important during times of great stress, like that experienced during the COVID-19 pandemic. Finally, incorporating families in the creation of policies should continue to be the standard of care. Even, and especially, in times of rapid change and great uncertainty, we need mechanisms for real-time feedback and input from families and opportunities for shared decision-making models of care. Figure 2 illustrates these potential interventions.

## Limitations

Our findings are limited by the homogeneity of the sample. The majority of the respondent parents were white mothers. This may be a reflection of the parents who frequent and use NICU parent support groups on the internet. A quarter of respondents did not report on race or ethnicity. We explored potential demographic relationships to themes but observed no strong trends. It will be important to engage parents from minority communities when designing future research and policies to ensure their experiences are understood. Another limitation is that the anonymous online survey format, which prevents the ability for follow-up questions. While our study was available to a geographically diverse sample, we may have excluded parents who express themselves more comfortably orally than in writing. These methods also prevented follow-up contact with participants. Finally, the limited number of questions asked of the respondents creates the possibility that other important themes may not have been developed with the answers we received.

## Conclusion

This qualitative study included parents of infants requiring specialized care in the NICU during the COVID-19 pandemic. The descriptions of parent experiences document the emotional struggle of being separated from support systems, feelings of isolation, lack of family-centered care, and exacerbation of emotional distress already known to be common to the NICU journey. The experience of parents included intense and frequent disappointment at the system level of having their rights to be present with their infant restricted and desire for more empathy, validation, and inclusion in decision making. It is important to remember that the restrictions placed on parental presence and access to infants during the beginning of the COVID-19 pandemic were not made maliciously by hospital administrators. Rather, policy makers and care providers were forced to make important decisions with little information, and a great deal of responsibility for safety. Now that we know more, we must do better as we move forward. We have begun to understand the lived experiences of parents with infants in the NICU and we have an opportunity to shape future policy decision making processes, especially in times of crisis. Parents and families need to be considered fundamental in this process. The COVID-19 pandemic uprooted the lives of nearly everyone. For parents of infants requiring hospitalization in the NICU during this time, the pandemic exacerbated this already challenging experience. Moving forward, healthcare providers and researchers can use our results to focus assessments, offer supportive services and emotional support, and remain steadfast in valuing the essential role of parents, when families encounter an infant hospitalization.

## Supplementary Information

**Additional file 1: Supplemental Table 1**. Open ended questions.

**Additional file 2: Supplemental Table 2**. Codebook.

## Data Availability

The datasets used and/or analyzed during the current study are available from the corresponding author on reasonable request.

## Abbreviations

NICU: Neonatal Intensive Care Unit
