# Supplementary material for: Parents’ pandemic NICU experience in the United States: a qualitative study
Source: BMC Pediatr. 2021 Dec 9;21:558. doi: 10.1186/s12887-021-03028-w (PMC8655088; doi:10.1186/s12887-021-03028-w)
Supplement: Supplementary file 1 — Additional file 1: Supplemental Table 1. Open ended questions. [file 12887_2021_3028_MOESM1_ESM.docx]

Supplemental Table 1: Open ended questions

1. *We would like to know more about your interactions with the healthcare providers in the NICU during the COVID-19 Pandemic. If applicable, was there anything they could have done differently?*
2. *How has the COVID-19 Pandemic impacted your transition home?*
3. *If you have more you would like to share with us about your experience or this survey, please share here.*
4. *Do you believe your experience of having a baby in the NICU was changed because of the COVID-19 Pandemic? Please share more about how your birth experience was impacted by the COVID-19 Pandemic.*
5. *Please share with us what your visitation experience was like in the NICU as it relates to the COVID-19 Pandemic.*
