# Supplementary material for: Parents’ pandemic NICU experience in the United States: a qualitative study
Source: BMC Pediatr. 2021 Dec 9;21:558. doi: 10.1186/s12887-021-03028-w (PMC8655088; doi:10.1186/s12887-021-03028-w)
Supplement: Supplementary file 2 — Additional file 2: Supplemental Table 2. Codebook. [file 12887_2021_3028_MOESM2_ESM.docx]

Supplemental Table 2: Codebook

| Name | Description |
| --- | --- |
| COVID vs. NICU | Topical code for responses that explicitly compare NICU during COVID with NICU in other times. |
| Finances | Discussion of impact of COVID on family finances. |
| Positive experience due to COVID | Descriptions of positive experiences due to COVID (e.g., parking access). |
| Work, work loss issues | Pandemic-related work or work loss issues. |
| Emotional, mental experience | Parent code for emotional and mental experiences. |
| Anger and frustration | Expressions of the spectrum from annoyance to frustration to anger. |
| Compounded experiences | Descriptions of cumulative impacts of emotional and mental experiences. |
| Depression, PPD, other mental illness | Depression, post-partum depression, and other diagnosed mental illnesses. |
| Disconnect, strange, cold, IRL | Expressions of disconnection, strangeness, and social coldness resulting from masking, social distance, isolation, and virtual interactions in contrast to “IRL” (in real life). |
| Exhaustion and sleep loss | Tiredness, exhaustion, sleep deprivation. |
| Fear, anxiety, panic | Expressions of the spectrum from anxiety to fear to panic. |
| Fear of COVID, illness | Fears specific to contracting COVID or other illnesses. |
| Gratitude | Expressions of gratitude, appreciation. |
| Grief, sadness, heartbreak, bittersweet | Expressions of the spectrum from “bittersweet” to sadness to grief to heartbreak. |
| Isolation, loneliness, missing | Talk of isolation, loneliness, or missing people. |
| Lacking emotional support, partner | Impacts of lacking emotional support, including in particular from partner’s absence due to restrictions. |
| Psychotherapy | Discussion of professional psychotherapy. |
| Rights violated | Talk of one’s rights being violated. |
| Stress, difficulty, hard, taxing, impossible, overwhelm | Expressions along the spectrum from difficulty to stress to impossibility and overwhelm. |
| Trauma | Explicit discussion of emotional or mental trauma. |
| Uncertainty | Discussion of uncertainty as contributing to emotional stress. |
| Implications of visitor restrictions | Parent code for all discussion of implications of visitor restrictions. (Also see “Visitor restrictions logistics” for strictly logistical topics.) |
| Breastfeeding needs | Restrictions in interaction with breastfeeding. |
| Burden on birth mother | Restrictions causing additional emotional, mental, and physical burdens on the birth parent. |
| Consult, class, learning implications | Restrictions impacting access to provider consults, classes, and other learning opportunities. |
| Essential caregivers not visitors | Assertions that parents should be considered essential members of the caregiving team, not be subject to the same visitor restrictions as other hospital visitors. |
| Forced choices | Discussion of restrictions forcing families to make difficult choices (e.g., which parent can visit infant, dividing parental attention between infant and older children, work/family choices, etc.) |
| Lost experiences w infant | Parent code for visitor restrictions resulting in parents losing experiences with infants they might otherwise have had. |
| Family unit | Restrictions preventing opportunities for the family to be together as a whole unit (e.g., both parents, older siblings, with infant) |
| Firsts | Lost opportunities to witness infant’s “firsts” (e.g., first bath, first feeding, etc.) |
| Kangaroo Mother Care (KMC), skin-to-skin | Restrictions preventing opportunities to give infants KMC or skin-to-skin care. |
| Meeting others | Restrictions preventing other friends and family from meeting infant. |
| Parental bonding | Restricted opportunities for parents to bond with infant. |
| Photos | Restricted opportunities to take professional photos, maskless photos, or photos of the family as a whole unit. |
| Seeing face and smiles, masking | Discussion of masking and lost opportunities for infant to see full faces and smiles. |
| Sharing with partner | Missed opportunities to share particular experiences with the infant with the partner, e.g., infant milestones, bad news, consults. |
| Touching, holding, kissing | Restricted opportunities to touch, hold, or kiss infant. |
| Need more support | Talk of needing more unspecified support than available due to restrictions. Also see “Lacking emotional support.” |
| Nonsensical policies | Talk of visitor policies being nonsensical, illogical, contradictory, etc. |
| Perceptions of time, long separations | Any discussion evocative of length of separations or other time periods (e.g., time in NICU) as burdensome due to restrictions. |
| Relationship btw parents | Impact of restrictions on the relationship between parents. |
| Travel and waiting | Impact of restrictions on necessary travel and waiting. |
| Twin implications | Implications of restrictions for twin infants, e.g., both parents permitted to visit (one per infant) or only one parent permitted despite two infants. |
| Staff interactions | Parent code for discussion of interactions with staff |
| Advocacy | Talk of parents advocating for patient or family with staff. |
| Communication issues | Discussion of communication with staff. |
| Access to doctors | Talk of access to speak to doctors, or lack thereof. |
| Contradictory btw staff | Talk of contradictory messaging from staff. |
| Healthcare treatment issues | Discussion of infant’s healthcare treatment, medical issues, etc. |
| Lactation support | Staff support for lactation needs. |
| PPE | Talk of staff use of Personal Protective Equipment |
| Professionalism, confidentiality | Talk of staff exhibiting professionalism and confidentiality, or lack thereof. |
| Remote interactions | Remote interactions with staff, e.g., phone, videoconference. |
| Rounds | Mention of experiences with staff rounds or ability to be present during rounds. |
| Staff changes, rotations | Talk of staff changes, including rotations, and the impact on patient and family. |
| Staff screening, hygiene | Discussion of staff COVID screening and COVID hygiene issues. |
| Staffing shortages | Staff shortages, usually attributed to COVID, and impacts on patient. |
| Support, validation, empowerment | Talk of staff providing support, validation, and empowerment to parents. |
| Unkindness, undermining, no compassion | Talk of parents experiencing staff as unkind, lacking in compassion, or undermining them. |
| Visitor restrictions logistics | Parent code for visitor restrictions logistics only. (See “Implications of visitor restrictions” for impacts of restrictions.) |
| Changing policies | Code for talk of visitor policies changing. |
| During infant procedure | Visitation restricted when infant is undergoing a procedure. |
| During mother's hospitalization | Restrictions on visitation of birth parent during their pre- or post-birth hospitalization. |
| Excluding grandparents | Talk of policies excluding grandparents. |
| Excluding misc | Talk of policies without specifying who is excluded. |
| Excluding siblings | Talk of policies excluding the infant’s older siblings. |
| Hours restricted | Talk of policies restricting visitation hours. |
| Hours unrestricted | Mention that policies did not restrict hours. |
| Mother only | Policies permitting only mothers. |
| No visitors | Policies prohibiting all visitors. |
| Number of visitors | Talk of policies limiting visitors by number at a time. |
| Number of visits | Talk of policies limiting by number of visits per day. |
| One caregiver at a time | Talk of policies permitting only one caregiver at a time. |
| One designated parent only | Talk of policies permitting only one designated parent visitor. |
| One person at a time | Talk of policies permitting only one person at a time. |
| Parents only | Talk of policies permitting both parents only. |
| Restricted reentry or exit | Talk of policies that restrict visitor exit or re-entry. |
| Restricted switch-offs | Talk of policies that restrict switches between visitors: |
| 24 hour switch-offs | Switch-offs permitted only once per 24-hour period. |
| Weekly switch-offs | Switch-offs permitted only once per 7-day period. |
| Scheduled visits | Policies that require scheduling of visits and visitors. |
| Exemplary quotes | Code to highlight exemplary quotes. |
